# Shear displacement gradient in X-ray Bragg coherent diffractive imaging

**DOI:** 10.1107/S1600577522002363

**Published:** 2022-04-05

**Authors:** Oleg Gorobtsov, Andrej Singer

**Affiliations:** a Department of Materials Science and Engineering, Cornell University, 418 Thurston Hall, Ithaca, NY 14853, USA

**Keywords:** coherent X-ray imaging, nanocrystals, crystal defects

## Abstract

Bragg coherent X-ray diffractive imaging is a cutting-edge method for recovering three-dimensional crystal structure with nanoscale resolution. Phase retrieval provides an atomic displacement parallel to the Bragg peak reciprocal lattice vector. The derivative of the displacement along the same vector provides the normal strain field, which typically serves as a proxy for any structural changes. In this communication it is found that the other component of the displacement gradient, perpendicular to the reciprocal lattice vector, provides additional information from the experimental data collected from nanocrystals with mobile dislocations. Demonstration on published experimental data show how the perpendicular component of the displacement gradient adds to existing analysis, enabling an estimate for the external stresses, pinpointing the location of surface dislocations, and predicting the dislocation motion in *in situ* experiments.

## Abstract 2

Experimental data are used to extract additional information on external stresses and surface dislocations through the perpendicular component of the atomic displacement gradient retrieved with Bragg coherent diffractive imaging.

## 1. Introduction

Imaging three-dimensional displacement and strain in nanocrystals using X-ray Bragg coherent diffraction imaging (BCDI) (Robinson *et al.*, 2001 ▸; Vartanyants & Robinson, 2001 ▸; Pfeifer *et al.*, 2006 ▸) enables physical discoveries in nano­structured materials from catalyzers to batteries. In a BCDI experiment, one measures a three-dimensional (3D) intensity in the reciprocal space containing the coherent *hkl* Bragg diffraction peak. An iterative algorithm then solves the phase problem, retrieving the shape of a crystal and the 3D atomic displacement field projected onto the reciprocal lattice vector **Q**_*hkl*_ that corresponds to the Bragg peak (Pfeifer *et al.*, 2006 ▸; Williams *et al.*, 2003 ▸). The method typically has a spatial resolution of tens of nanometres, sufficient to reveal the defect signatures and the crystal deformation.

**Figure 1:**
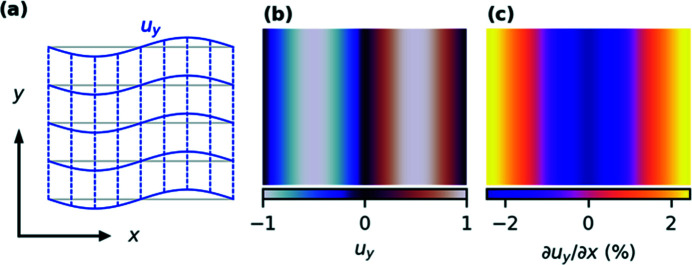
Schematic of the displacement and its gradient. (*a*) A schematic representation of a displacement field inside a crystal (blue) with respect to an unperturbed crystal lattice (gray). The momentum transfer **Q** is parallel to the *y*-axis, and the experiment is insensitive to the vertical crystal planes (dashed lines). (*b*) The 2D displacement field *u*_*y*_(*x*,*y*) in false color, typically directly extracted from BCDI data through phase retrieval. We chose the field such that the normal strain ∂*u*_*y*_/∂*y* = 0. (*c*) The displacement gradient ∂*u*_*y*_/∂*x* in the direction perpendicular to **Q** clearly shows the bending of the planes visible in (*a*). In (*b*) and (*c*) the *x* and *y* coordinates are identical to those in (*a*).

Fig. 1 ▸(*a*) schematically shows a region of a deformed model crystal. Then reciprocal space measured around the *hkl* peak with BCDI only records displacement of the (*hkl*) planes along the scattering vector {here in Fig. 1 ▸(*a*), direction *y* is defined as along the [*hkl*] direction}, *i.e.* the direction normal to the planes of the undeformed crystal (Robinson *et al.*, 2001 ▸; Vartanyants & Robinson, 2001 ▸). The displacement field is *u*_*y*_(*x*, *y*) = *r*(*x*, *y*) − *r*_0_(*x*, *y*), where *r*(*x*, *y*) are the positions of the distorted planes and *r*_0_(*x*, *y*) are the positions of the undistorted planes. The displacement along the *y*-direction is shown in false color in Fig. 1 ▸(*b*). The direct interpretation of the displacement field is often complicated by a linear slope that arises from imprecise centering of the Bragg peak due to the discrete data collection. Instead, one often interprets the gradient of the displacement field in the direction of the scattering vector **Q**_*hkl*_. Taking the derivative of a linear variation in displacement returns a constant strain, and, by definition, ɛ_*yy*_ = ∂*u*_*y*_/∂*y* is the normal strain in the *y*-direction, which is the local variation in the lattice constant.

While the atomic displacement field and the normal strain field are often calculated and interpreted in BCDI experiments (Singer *et al.*, 2018*a* ▸; Ulvestad *et al.*, 2015*a* ▸,*b* ▸), the component of the displacement field gradient perpendicular to the scattering vector is seldom considered. This excludes useful information on the variation of the atomic displacement along the crystallographic planes. It has been suggested that the transverse derivative of the displacement field can be a measure of the plane disorder (Kawaguchi *et al.*, 2019 ▸), or can be used to help define the full strain tensor in multireflection experiments (Hofmann *et al.*, 2017 ▸, 2020 ▸). Consider that in the example in Fig. 1 ▸ the crystal deformation includes bending of the crystallographic planes without changing their relative spacing. The normal strain [derivative along *y* in Fig. 1 ▸(*b*)] vanishes, consistent with the notion that pure bending can leave the lattice constant perpendicular to the bending unchanged. Nevertheless, the bending of the crystallographic planes is still clearly visible in the perpendicular component of the displacement gradient ∂*u*_*y*_/∂*x*. The displacement derivative ∂*u*_*y*_/∂*x* is a part of the shear strain ɛ_*yx*_, defined symmetrically as ɛ_*yx*_ = 1/2(∂*u*_*y*_/∂*x* + ∂*u*_*x*_/∂*y*). Because BCDI on a single peak only records *u*_*y*_, no direct measurement of the shear strain is possible unless multiple peaks are recorded to extract the full strain tensor (Hofmann *et al.*, 2017 ▸, 2020 ▸; Newton *et al.*, 2014 ▸). The challenge to record multiple peaks exists due to the geometrical limitations such as the small crystal and X-ray focus size, specifically in operando multicomponent systems. Here, we consider the component of the displacement gradient ∂*u*_*y*_/∂*x* (hereafter referred to as shear gradient) for interpretation of BCDI experiments, in the absence of access to the full strain tensor. We present an experimental example of using the shear gradient to derive hitherto undiscussed properties from BCDI data, specifically the propagation direction of dislocations in nanocrystals.

## 2. Results and discussion

**Figure 2:**
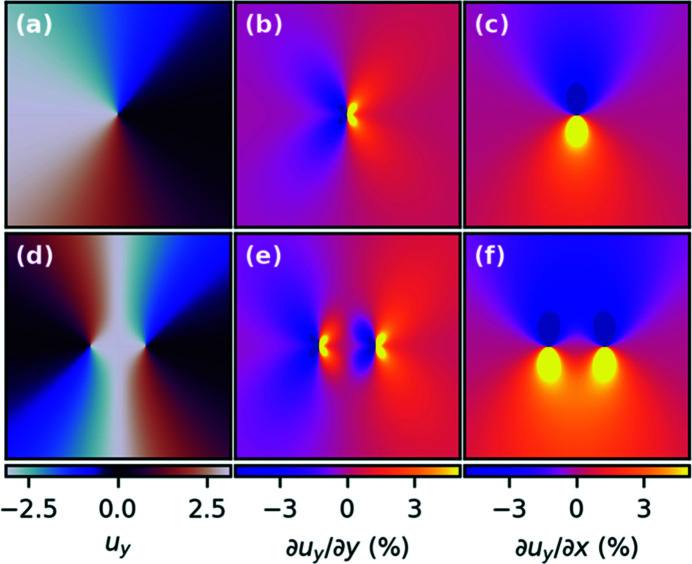
Displacement and its gradient in the presence of crystal defects. (*a*) The displacement field around an edge dislocation with the Burgers vector oriented vertically and the extra half plane inserted from the left. (*b*) The normal strain ∂*u*_y_/∂*y* and (*c*) the displacement gradient perpendicular to **Q**, ∂*u*_*y*_/∂*x*. (*d*–*f*) The displacement field and gradients for a pair of dislocations.

BCDI has recently proven itself as an effective tool to image dislocations, which play a key role in the chemical and structural properties of functional materials (Ulvestad *et al.*, 2015*b* ▸; Clark *et al.*, 2015 ▸; Singer *et al.*, 2018*b* ▸; Sun *et al.*, 2021 ▸; Jacques *et al.*, 2011 ▸; Dupraz *et al.*, 2017 ▸). Three types of dislocations exist: screw dislocations, edge dislocations, and mixed dis­locations. Within continuum mechanics, the displacement fields along the Burgers vector are given by (Hirth & Lothe, 1982 ▸)

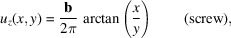




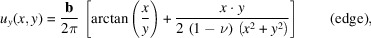

where **b** is the Burgers vector and ν is the Poisson ratio. For a screw dislocation, the Burgers vector and the sense vector (tangential to the dislocation line) are parallel: *b*∥*z*. For an edge dislocation both are perpendicular, 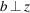 . At the dis­location, the Burgers circuit construction – a contour integral over the displacement gradient around the dislocation – yields a non-vanishing Burgers vector equal to the corresponding plane spacing. As a result, the displacement field contains a singularity and a surrounding vortex on the plane perpendicular to the dislocation line. Fig. 2 ▸ shows this singularity for an edge dislocation [Fig. 2 ▸(*a*)]. If in a BCDI experiment the scattering vector **Q**_*hkl*_ has a non-vanishing component along the Burgers vector, the retrieved displacement field will also display a singularity. An edge is visible on the plane containing the **Q**_*hkl*_ vector, while a screw is visible on the plane normal to the **Q**_*hkl*_ vector.

The displacement gradient components for screw and edge dislocations can be derived analytically (Hirth & Lothe, 1982 ▸) and both are shown in Figs. 2 ▸(*b*) and 2(*c*). These quantities can be directly calculated from the experimentally retrieved 3D displacement field by taking the derivative numerically. For a screw dislocation, both the normal and the shear gradient are identical, simply rotated by 90° (not shown here). For an edge dislocation, normal strain and shear gradient show different signatures of the dislocation. The shear gradient is more spread out spatially and is larger in magnitude (see Fig. 2 ▸), and its extension away from the dislocation line strongly depends on the Poisson ratio. A comparison between the normal and shear gradients therefore allows us to study the Poisson ratio, similar to the analysis reported by Ulvestad *et al.* (2015*b* ▸). Additionally, the shear gradient at a distance from the dis­location [see Fig. 2 ▸(*d*)] is slightly larger in magnitude than the normal strain. This is particularly useful for studying dislocations at the grain boundary since the displacement field in the proximity of the grain boundary is generally difficult to recover with BCDI. Usually, BCDI is more sensitive to probe the structure in the bulk, and the boundary is determined from the retrieved amplitude by setting an arbitrary threshold between 0.1 and 0.5, and thus capturing the dislocations at the grain boundary is unreliable. Therefore, having a displacement gradient reaching deeper inside the grain can increase the sensitivity of BCDI to study dislocations located at the grain boundary.

In addition to enabling an extended dislocation imaging, the shear displacement gradient offers a way to gain further insight into other stress contributions present in the specimen. These other stresses can arise from the presence of neighboring dislocations in the specimen, external stresses, or image forces (Hirth & Lothe, 1982 ▸) on dislocations nucleating near the crystal surface. Image forces arise because the stress at the interface vanishes and can be understood as a force from an oppositely oriented imaginary dislocation placed on the opposite side of the interface. Notably, shear stresses oriented along the Burgers vector result in dislocation glide. The shear displacement gradient is a combination of a rigid body rotation and shear strain: one cannot extract the shear strain from the displacement field along a single direction measured in single-peak BCDI. Nevertheless, if the inhomogeneity in the shear gradient emerges in combination with observed dislocation motion, it can serve as evidence to estimate the mechanisms behind the evolution.

**Figure 3:**
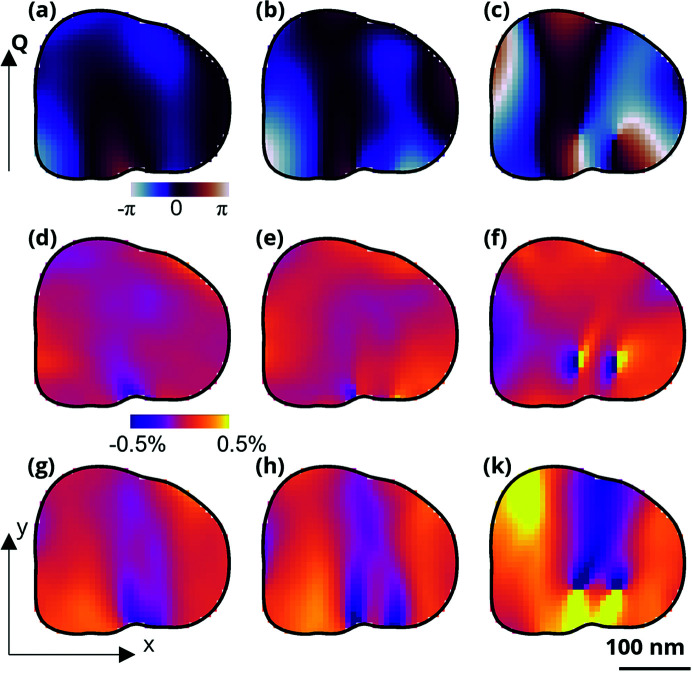
Shear gradient calculated from the operando imaging data experiment described in Singer *et al.* (2018*a* ▸). (*a*–*c*) Cross-sections of the displacement field within a single grain at different stages of the battery charging. (*d*–*f*) Strain field, ∂*u*_*y*_/∂*y*. (*g*–*k*) Displacement gradient ∂*u*_*y*_/∂*x* perpendicular to the **Q** vector proposed in this work. Charge states 1 (*a*, *d*, *g*), 2 (*b*, *e*, *h*), and 3 (*c*, *f*, *k*) correspond to charging a lithium-rich layered oxide particle at voltages of 4.0 V, 4.2 V, and 4.3 V, respectively.

We further demonstrate the new insights provided by the shear displacement gradient by re-analyzing the operando experimental data published by Singer *et al.* (2018*a* ▸). This study reported operando dislocation formation, which was correlated with the voltage fade in a lithium-rich layered oxide material for high-capacity electrodes in lithium-ion batteries. In the paper, the authors measured the atomic displacement *u*_*y*_(*r*) along the 002 reciprocal lattice vector and calculated the normal strain field ∂*u*_*y*_(*r*)/∂*y*, both reproduced in Figs. 3 ▸(*a*)–3(*f*). Dislocations are recognized in the displacement field as singularities. The displacement field at charge states 1 and 2 display no singularities. At charge state 3, two singularities are visible in the displacement field. Based on the direction of the Burgers vector in comparison with the scattering vector, they correspond to two edge dislocations that formed between charge states 2 and 3. The dislocations are also visible in the normal strain showing compressive and tensile strain on the opposite sites of the dislocation. From the normal strain maps [see Figs. 3(*d*)–3(*f*) ▸], the authors hypothesized that the tensile strain build-up at the lower right section of the image that emerges at charge state 2 possibly leads to the generation of defects.

Going beyond what was reported by Singer *et al.* (2018*a* ▸), we calculate the shear displacement gradient ∂*u*_*y*_(*r*)/∂*x* [see Fig. 3 ▸(*c*)]. The direction *x* is perpendicular to the Burgers vector and the dislocation line of the observed edge dislocations (unique direction). The dislocations are visible at charge state 3 as vertical bands of high positive and negative shear gradient above and below both dislocations. Interestingly, while the normal strain shows no apparent features in charge states 1 and 2, the shear displacement gradient is highly inhomogeneous in charge states 1 and 2. Specifically, inhomogeneities appear in the region where the dislocations later emerge. The shear displacement gradient at charge states 1 and 2 before dislocation nucleation reveals a narrow vertical band, about 100 nm wide, where the shear gradient lowers and raises again. In charge state 2, the shear bands are visible in the particle indicating incipient dislocations at the grain boundary. Similar to the one shown in the schematic in Fig. 1 ▸(*c*), the experimentally found shear gradient reveals an undulation in the crystalline layers [see Fig. 1 ▸(*a*)]. Additionally, these shear bands visible before the dislocation formation predict where the dislocations will emerge: the dislocations move along the direction of that shear band. Because the undulations are visible before the dislocations occur, they likely arise because of other stresses present in the particle. The measured particle is surrounded by other particles agglomerated tightly into a secondary particle. The anisotropic lattice changes inside the neighboring particles is therefore a possible reason for the external stress on the measured particle.

Now we will attempt to interpret the dislocation motion from the displacement gradient. An edge dislocation moves within the slip plane (parallel to the Burgers vector) following the force per unit length given by the Peach–Koehler equation *f* = τ_*xy*_ *b*. Here, τ_*xy*_ is the shear stress resolved onto the slip plane where the dislocation moves. Because the dislocation moves along the undulation band, it is plausible that this stress is the origin of dislocation formation at the boundary and its motion into the bulk. We estimate the shear stress through Hooke’s law, τ_*xy*_ = *G*ɛ_*xy*_. The shear modulus *G* of LiCoO_2_ (structurally similar to the material studied here) is of the order of 100 GPa (Qi *et al.*, 2014 ▸) in the discharged (li­thia­ted) state and 30 GPa in the charged (deli­thia­ted) state. By assuming that the value of the full symmetrically defined shear strain ɛ_*xy*_ = (1/2)(∂*u*_*y*_/∂*x* + ∂*u*_*x*_/∂*y*) is similar in magnitude to the displacement gradient ∂*u*_*y*_/∂*x* we measure (note that ∂*u*_*y*_/∂*x* and ∂*u*_*x*_/∂*y* are not equal generally, so this is an order of magnitude estimate at best), we estimate the shear stress. The value of ∂*u*_*y*_/∂*x* from Figs. 3 ▸(*g*)–3(*i*) is smaller than 0.01, yielding a shear stress of 1 GPa for the li­thia­ted and 0.3 GPa for the deli­thia­ted material. Combining this result with the value for the Burgers vector of 5 Å, measured in Singer *et al.* (2018*a* ▸), we estimate the force per unit length of dislocation of *f* = 0.25 N m^−1^, and the total force on the dislocation of *F* = *f* *L* = 50 nN (*L* = 200 nm is the length of the dislocation).

The Peierls stress is the fundamental property that resists dislocation motion. In Fig. 3 ▸, we observe that the dislocation only starts moving when the shear stress increases above the estimated value, suggesting that the shear stress we estimate from the displacement gradient is comparable in magnitude with the Peierls stress. The value we find (1 GPa) for the gradient is about an order of magnitude smaller than in the covalently bonded ceramics and is comparable with semicovalent and ionic bonded ceramics (Kamimura *et al.*, 2013 ▸).

## 3. Conclusions

In summary, we show that the displacement field determined in X-ray Bragg coherent diffractive imaging experiments from a single Bragg peak contains additional beneficial information on the shear strain components. We calculate the displacement gradient perpendicular to the scattering vector and demonstrate that this gradient includes information beyond the normal strain, which is usually discussed in the literature. We applied the shear displacement gradient to previously published experimental operando data, which discussed dislocation nucleation in battery nanoparticles during charge. Our result allows detection of dislocations at the crystal surface and predicts the path of dislocations movement. Additionally, we use the magnitude of the shear gradient to estimate the Peierls stress in operando experiments on nanoparticles.
